# Investigation of the In-Plane Mechanical Anisotropy of Magnesium Alloy AZ31B-O by VPSC–TDT Crystal Plasticity Model

**DOI:** 10.3390/ma12101590

**Published:** 2019-05-15

**Authors:** Bo Zhang, Shuangming Li, Huamiao Wang, Weiqin Tang, Yaodong Jiang, Peidong Wu

**Affiliations:** 1 School of Mechanics & Civil Engineering, China University of Mining and Technology (Beijing), Beijing 100083, China; zbdcumtb@163.com (B.Z.); jiangydcumtb@163.com (Y.J.); 2 State Key Laboratory of Solidification Processing, Northwestern Polytechnical University, Xi’an 710072, China; lsm@nwpu.edu.cn; 3 State Key Laboratory of Mechanical System and Vibration, Shanghai Jiao Tong University, Shanghai 200240, China; Weiqingtangx0@126.com; 4 Department of Mechanical Engineering, McMaster University, Hamilton, ON L8S 4L7, Canada; peidong@mcmaster.ca

**Keywords:** AZ31B alloy, anisotropy, polycrystal plasticity, twinning, R-value

## Abstract

The in-plane mechanical anisotropy of magnesium alloy sheet, which significantly influences the design of the parts produced by Mg alloy sheets, is of great importance regarding its wide application. Though the stress–strain response and texture evolution have been intensively investigated, and the anisotropy of Mg alloy can be significantly substantiated by its R-value, which reveals the lateral response of a material other than the primary response. As a consequence, the conjunction of viscoplastic self-consistent model and twinning and detwinning scheme (VPSC–TDT) is employed to investigate the in-plane anisotropy of magnesium alloy AZ31B-O sheet. The loading cases include both tension and compression along different paths with respect to the processing direction of the sheet. It is revealed that the stress–strain relation, texture evolution, R-value, and involved deformation mechanisms are all loading path-dependent. The unique R-values of Mg alloys are interpreted with the aid of modeling behaviors of Mg single crystals. The results agree well with the corresponding experiments. It is found that the hexagonal close-packed (HCP) crystallographic structure, deformation twinning, and initial basal texture are responsible for the characteristic behavior of Mg alloys.

## 1. Introduction

Due to the low symmetry of hexagonal close-packed (HCP) crystal structure and the existence of a twinning mechanism, magnesium alloy usually exhibits strong anisotropic behavior [1,2,3,4,5]. For instance, strong asymmetry in stress–strain responses between tension and compression, and large hysteresis loops under cyclic loading, have been observed for wrought Mg alloy rolled sheets or extruded bars [6,7,8,9,10]. The different mechanical behaviors over a wide range of strain rates and temperatures along different loading directions have revealed the strong anisotropy of AZ31B Mg alloy [6,7]. Significant anisotropic behavior of Mg alloys has been manifested in terms of the strong tension–compression asymmetry [8]. Unlike tension and compression, the nearly symmetrical shear stress–shear strain curve was ascribed to prismatic slips and the simultaneous activation of deformation twinning and detwinning under reverse shear [9]. The texture-induced strength anisotropy and asymmetry as well as anisotropic strain hardening behavior was investigated through the twinning and detwinning (TDT) model [10]. These behaviors influence the design of parts produced by Mg alloys. Based on the primary stress and strain components (with respect to loading direction), the in-plane anisotropy of Mg alloy sheets was not believed to be significant due to the not-so-large asymmetry of their initial texture [11,12,13]. However, the in-plane anisotropy could be better evaluated by additionally considering the lateral stress or strain components. R-value, which is the ratio of the lateral strain components, is an important parameter in constitutive modeling of the anisotropic behavior of sheet metals. Many constitutive models have employed R-values as material constants to better describe the materials’ anisotropic behaviors [14,15].

Most of the studies on the R-value of Mg alloy sheets are under in-plane tension. It is found that tensile R-values increase significantly (>1) with straining, which are attributed to the strong basal texture and HCP crystallographic structure [11,16,17,18]. With respect to in-plane compressions, the R-values are usually less than 1 [11,16,19], and even negative values have been observed [20,21], which was attributed to the activity of extension twin. However, it is still unclear if the activation of extension twin will certainly induce negative compressive R-values. Since R-values can characterize the formability of materials, the inconsistency found in literature demands further investigation of the R-values of Mg alloy sheets. As a consequence, this work will carefully study the in-plane anisotropy of Mg alloy sheets under monotonic loadings along different directions in terms of the R-value, which will significantly substantiate the in-plane anisotropy of Mg alloy sheets.

Due to its computational efficiency compared to full field models, mean-field crystal plasticity models are widely applied [13,22,23,24,25]. It has been demonstrated that the self-consistent models were more appropriate for Mg alloys with HCP crystallographic structure among the mean-field models [26,27,28]. The used models are the viscoplastic self-consistent polycrystal model (VPSC) proposed by Lebensohn and Tomé [13] and the elastic–plastic self-consistent model (EPSC) proposed by Turner and Tomé [29]. In order to account for both the rate sensitivity and elasticity of a material, Wang et al. developed a finite strain elastic–viscoplastic self-consistent (EVPSC) model for polycrystalline materials [30]. In order to accurately capture the mechanical behavior of Mg alloys, deformation twinning has to be considered [31]. Therefore, the twinning scheme implemented in the mean-field model is critical for the accurate description of the mechanical behaviors of Mg alloys. The predominant twin reorientation (PTR) [32], volume fraction transfer (VFT) [33], and composite grain (CG) [34,35] schemes were developed by Tomé and his coworkers. Kalidindi et al. [33,36] developed appropriate constitutive functions for slip–twin interactions, together with an efficient time-integration scheme. These models merely consider either the twinning-induced reorientation or the predominant twinning variant. In fact, an appropriate crystal plasticity model for describing both twinning and detwinning had not been developed until the work of Wang et al. [37]. The predictive capability of the twinning and detwinning model (TDT) has been demonstrated by applying it to magnesium alloys under both monotonic and cyclic loadings [38,39,40,41,42,43], where twinning and/or detwinning were best exhibited.

In this paper, the deformation behavior of AZ31B magnesium alloy sheet is investigated by the combination of the VPSC model and the TDT scheme (VPSC–TDT). The anisotropic behaviors of Mg alloy sheet are investigated in terms of stress–strain response, texture evolution, and the evolution of the R-values under various loading conditions. The modeling results are compared to the corresponding experiments.

## 2. Experiment

**Figure 1:**
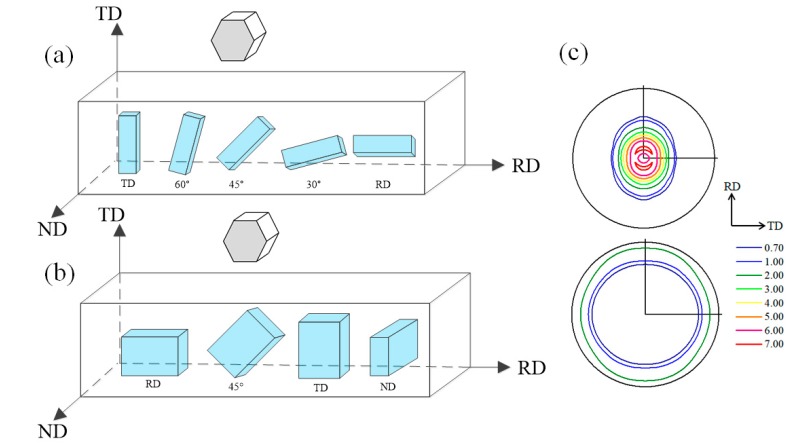
Schematics of (**a**) the tensile samples and (**b**) compressible samples, and (**c**) pole figures of the initial texture.

The studied material was 1.57 mm AZ31B-O sheet in an annealed (O temper) condition. The chemical composition of the alloy is listed in Table 1. Neutron diffraction was used to obtain the initial texture of the material. Tensile testing was performed on bone-shaped samples with 25 mm gage length. Five different orientations with tilt angles of 0, 30, 45, 60, and 90° with respect to (WRT) the rolling direction (RD) (Figure 1a) were used to study the mechanical anisotropy under uniaxial tension. Compressive tests were performed on the solid cube 8 mm on a side and conducted along four orientations, namely the RD, 45° WRT RD, transverse direction (TD), and normal direction (ND) (Figure 1b). All mechanical tests were carried out on an Instron 1331 (Instron, Boston, MA, USA) tension/compression servohydraulic load frame at ambient temperature (23 °C) and strain rate of 0.001 s^−1^. A more detailed depiction of the experimental process can be found in Tari et al. [19].

## 3. VPSC–TDT Model

The VPSC–TDT model is only briefly described here. Detailed descriptions can be found elsewhere [13,30,37]. The shear rate for both slip and twinning systems are expressed in the form of power law:

(1) $$\dot{\gamma }={\dot{\gamma }}_{0}{\left|\tau /\tau_{cr}\right|}^{1/m}\mathrm{sgn}\left(\tau \right)$$

where ${\dot{\gamma }}_{0}$ is a reference shear rate, τ is the resolved shear stress, $\tau_{cr}$ is the critical resolved shear stress (CRSS), and m is the strain rate sensitivity.

For both slip and twinning, the critical resolved shear stress (CRSS) $\tau_{cr}^{\alpha }$ is updated by

(2) $$\tau_{cr}^{\alpha }=\frac{d{\hat{\tau }}^{\alpha }}{d\Gamma }\underset{\beta }{\sum }h^{\alpha \beta }\left|{\dot{\gamma }}^{\beta }\right|$$

where $h^{\alpha \beta }$ is the latent hardening coupling coefficient, which empirically accounts for the obstacles on system α associated with system β. ${\hat{\tau }}^{\alpha }$ is the threshold stress and is given by

(3) $${\hat{\tau }}^{\alpha }=\tau_{0}^{\alpha }+\left(\tau_{1}^{\alpha }+h_{1}^{\alpha }\Gamma \right)\left(1-\exp\left(-\frac{h_{0}^{\alpha }}{\tau_{1}^{\alpha }}\Gamma \right)\right)$$

where $\tau_{0}$, $h_{0}$, $h_{1},$ $\tau_{0}+\tau_{1}$, and $\Gamma =\sum_{\alpha }\int \left|{\dot{\gamma }}^{\alpha }\right|dt$ are the initial CRSS, the initial hardening rate, the asymptotic hardening rate, the back-extrapolated CRSS, and the accumulated shear strain, respectively.

The twinning and detwinning (TDT) model [28] is used to describe twinning. Four potential operations have been introduced in the process of twinning and detwinning, i.e., (A) twin nucleation, (B) twin growth, (C) twin shrinkage, and (D) retwinning. The grain starts twinning by twin nucleation, and the associated control condition is

(4) $${\dot{\gamma }}^{\alpha A}=\{\begin{array}{ll} {\dot{\gamma }}_{0}{\left|\tau^{\alpha }/\tau_{cr}^{\alpha }\right|}^{1/m} & \tau^{\alpha }>0\\ 0 & \tau^{\alpha }\leq 0 \end{array}.$$

The twin grows by twin growth, which is defined as

(5) $${\dot{\gamma }}^{\alpha B}=\{\begin{array}{ll} -\dot{\gamma_{0}}{\left|\tau^{\alpha }/\tau_{cr}^{\alpha }\right|}^{1/m} & \tau^{\alpha }<0\\ 0 & \tau^{\alpha }\geq 0 \end{array}$$

Twin shrinkage, which shrinks the developed twin, is opposite to twin growth:

(6) $${\dot{\gamma }}^{\alpha C}=\{\begin{array}{ll} -\dot{\gamma_{0}}{\left|\tau^{\alpha }/\tau_{cr}^{\alpha }\right|}^{1/m} & \tau^{\alpha }<0\\ 0 & \tau^{\alpha }\geq 0 \end{array}$$

Retwinning causes twinning in twin, whose shear rate is defined as

(7) $${\dot{\gamma }}^{\alpha D}=\{\begin{array}{ll} {\dot{\gamma }}_{0}{\left|\tau^{\alpha }/\tau_{cr}^{\alpha }\right|}^{1/m} & \tau^{\alpha }>0\\ 0 & \tau^{\alpha }\leq 0 \end{array}$$

Twin nucleation and twin growth increase the twin volume fraction (TVF), while twin shrinkage and retwinning decrease it. The evolution of the TVF associated with the four operations are individually characterized by

(8) $${\dot{f}}^{\alpha A}=\frac{\left|{\dot{\gamma }}^{\alpha A}\right|}{\gamma^{tw}},\;{\dot{f}}^{\alpha B}=\frac{\left|{\dot{\gamma }}^{\alpha B}\right|}{\gamma^{tw}},\;{\dot{f}}^{\alpha C}=-\frac{\left|{\dot{\gamma }}^{\alpha C}\right|}{\gamma^{tw}},\;{\dot{f}}^{\alpha D}=-\frac{\left|{\dot{\gamma }}^{\alpha D}\right|}{\gamma^{tw}},$$

where $\gamma^{tw}$ is the characteristic twinning shear strain.

The evolution of TVF associated with twinning system α is calculated by

(9) $${\dot{f}}^{\alpha }=f^{0}\left({\dot{f}}^{\alpha A}+{\dot{f}}^{\alpha C}\right)+f^{\alpha }\left({\dot{f}}^{\alpha B}+{\dot{f}}^{\alpha D}\right),$$

where $f^{\alpha }$ is the volume fraction of twinned region, and $f^{0}=1-\sum_{\alpha =1}^{N}f^{\alpha }$ is the volume fraction of the untwinned matrix.

The threshold volume fraction $V^{th}$ that is used to turn off twinning is defined as

(10) $$V^{th}=\min\left(1.0,\;A_{1}+A_{2}\frac{V^{eff}}{V^{acc}}\right),$$

where $A_{1}$ and $A_{2}$ are two material constants. $A_{1}$ essentially controls the level of strain that a grain can undergo prior to the twinning mechanism beginning to undergo exhaustion. $A_{2}$ controls the rate at which this exhaustion takes place once it has begun. Two statistical variables, $V^{acc}$ and $V^{eff}$, are the weighted volume fraction of the twinned region and the volume fraction of twin terminated grains, respectively.

## 4. Results and Discussions

### 4.1. Mechanical Behavior

Specimens (Figure 1a,b) with loading directions oriented differently with respect to the processing direction of the magnesium alloy AZ31B sheet were fabricated for all tests. The initial texture of the sheet is typical rolled texture (see Figure 1c, the {00.1} and {10.0} pole figures), which is represented by 1928 discrete orientations for simulation. The mechanical behavior of the sheet at room temperature has been reported by Tari et al. [19], where in-plane samples were fabricated along different directions with respect to RD. Both tension and compression tests have been performed by using these samples, which are denoted in the form of the loading direction. For example, the tension along RD is denoted as T-RD, while compression along 45° with respect to RD is denoted as C-45. The plastic deformation is assumed to be accommodated by the basal ($\left\{0001\right\}\langle 11\bar{2}0\rangle$), prismatic ($\left\{10\bar{1}0\right\}\langle 11\bar{2}0\rangle$), and pyramidal <c + a> ($\left\{11\bar{2}2\right\}\langle 11\bar{2}3\rangle$) slips, as well as the extension twinning ($\left\{10\bar{1}2\right\}\langle 10\bar{1}1\rangle$). The single crystal elastic constants are taken from the handbook by Simmons and Wang [44]: C_11_ = 58.0, C_12_ = 25.0, C_13_ = 20.8, C_33_ = 61.2, and C_44_ = 16.6 (unit of GPa). The reference slip/twinning rate ${\dot{\gamma }}_{0}$ and the rate sensitivity m are defined as 0.001 s^−1^ and 0.05, respectively.

**Figure 2:**
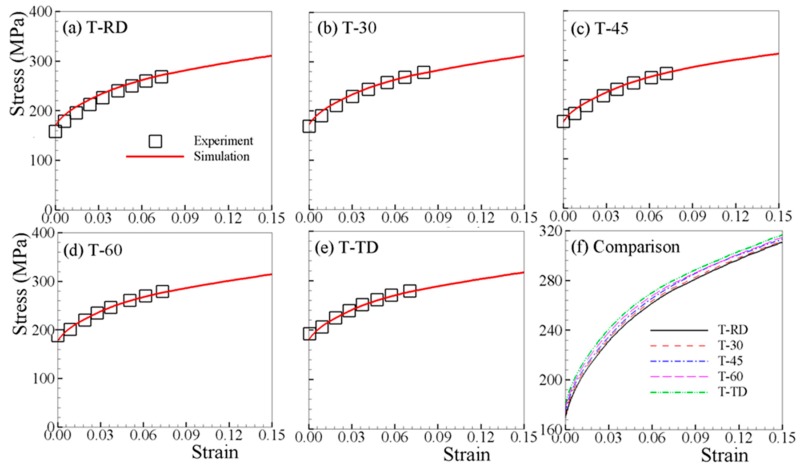
Measured and simulated stress–strain curves under uniaxial tension along different directions (**a**–**e**), and comparison of the simulated curves (**f**).

**Figure 3:**
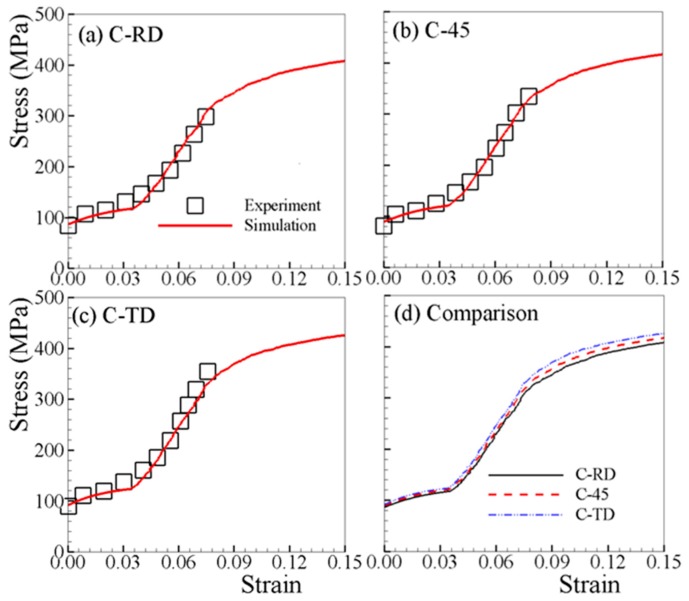
Measured and simulated stress–strain curves under uniaxial compression along different directions (**a**–**c**), and comparison of the simulated curves (**d**).

**Figure 4:**
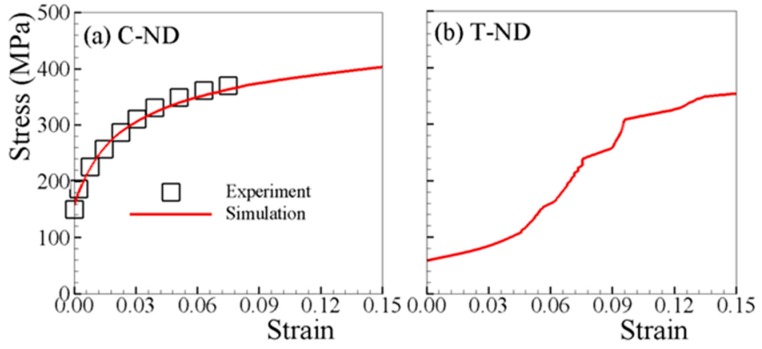
Measured and simulated stress–strain curves under uniaxial (**a**) compression (C-ND) and (**b**) tension (T-ND) along normal direction (ND).

The determined material parameters, through fitting both T-RD and C-RD stress–strain curves, are listed in Table 2. Figure 2a and Figure 3a show that a good match is obtained. The material parameters listed in Table 2 are used for all subsequent simulations. Figure 2 compares the experimental and predicted tensile stress–strain curves along RD; at 30, 45, and 60° with respect to RD; and TD. The yield stress increases slightly with the loading axis rotated from RD to TD. This is mainly due to the c-axis of grains in AZ31B sheet are oriented more towards RD than TD, though preferentially parallel to normal direction (ND) (Figure 1c). Figure 3 shows the stress–strain curves under uniaxial compression. It is obvious that the VPSC–TDT model can capture the experimental curves very well. The stress–strain behavior is dependent on the load orientation with respect to the rolling direction of the polycrystalline aggregate, suggesting the differences in operative deformation mechanisms in each of the five investigated orientations. From Figure 3, the stress–strain curves are S-shaped along all directions. Such a stress–strain tendency is consistent with the dominance of {10–12} twinning at low strains, exhibiting a region of low work-hardening rate up to ~3.5% plastic strain, followed by strong strain hardening at larger strains before a second yield phenomenon occurs, as has been typically and previously observed [39,40,45,46,47]. In general, AZ31B exhibits a lower stress level for a given strain in RD compared to the other directions for both tension and compression. Figure 4a,b depict the tensile and compressive stress–strain curves along ND, where distinct stress–strain curves are observed due to the difference in the twinning activity. It should be noted that stress jumps of the stress–strain curve of T-ND are not numerical, but due to the termination of twinning.

**Figure 5:**
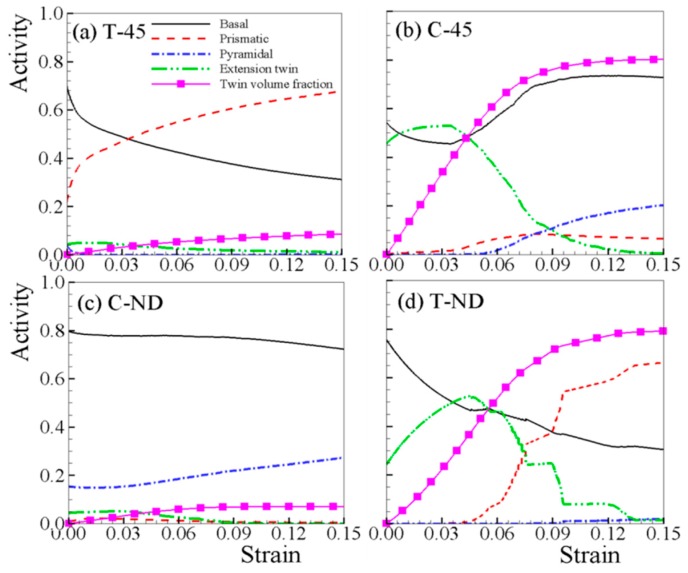
Relative activities for (**a**) tension along 45° (T-45), (**b**) tension along 45° (C-45), (**c**) compression along ND (C-ND), and (**d**) tension along ND (T-ND).

The relative activities of various deformation mechanisms under T-45 and C-45 are presented in Figure 5, since those under in-plane tensions (or in-plane compressions) are only qualitatively different. The plastic deformation under T-45 is dominated by basal and prismatic slip (Figure 5a). With increasing strain, basal slip becomes less active while prismatic slip increases steadily. The activity of pyramidal slip is very low throughout the straining. The activity of extension twin is steadily maintained until 3% plastic strain, and then decreases slightly with straining. For C-45 (Figure 5b), the initial plastic deformation is dominated mostly by basal slip and extension twin, and further deformation is mainly accommodated by prismatic and pyramidal slip. When the activity of extension twin drops sharply at 6% plastic strain, the activity of pyramidal slip starts to rise noticeably for coordinating c-axis deformation. The $\left\{10\bar{1}2\right\}$ twin volume fraction (TVF) reaches above 80%, while that under T-45 is less than 10%. For C-ND (Figure 5c), the plastic deformation is mostly dominated by basal and pyramidal slip, with little twinning in the ND compression samples. The deformation mechanisms of T-ND (Figure 5d) are similar to the C-45 (Figure 5b) during initial straining, where basal slip and extension twin are dominant. However, the activity of prismatic slip is much larger under T-ND than C-45, when the activity of extension twin decreases sharply.

**Figure 6:**
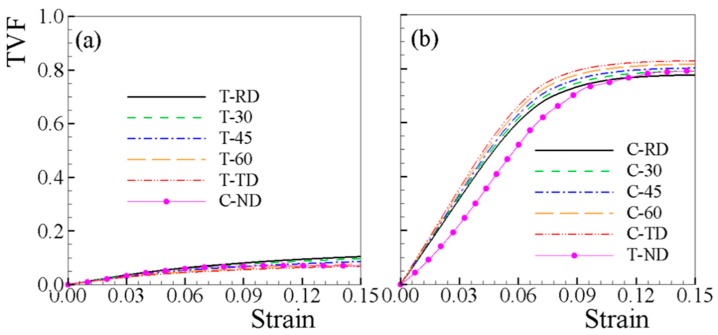
The predicted twin volume fraction as a function of strain under (**a**) twin-unfavorable and (**b**) twin-favorable loadings.

The evolution of the TVF under tension and compression is shown in Figure 6. The {0002} pole figures in Figure 1c shows a slight off-basal character of the primarily basal texture, which leads to the in-plane anisotropic behavior. In the T-RD, the $\left\{10\bar{1}2\right\}$ TVF is saturated at 11%. Regarding tension, the larger the loading direction tilts with respect to RD, the lower the $\left\{10\bar{1}2\right\}$ TVF (Figure 6a). The TVFs under compressive tests—which are apparently larger than in tensile samples and saturate at ~80%—endure an opposite tendency (Figure 6b). A slight off-basal shift of the c-axis of grains towards RD leads to more twinning under C-TD than C-RD. The TVF increases with tilting of the loading direction from RD to TD. In Figure 6, the TVFs of C-ND and T-ND are plotted together with other loadings. As shown in Figure 6a, the TVF of C-ND stabilizes early at ~7% strain due to the strongly basal texture.

### 4.2. Texture Evolution

**Figure 7:**
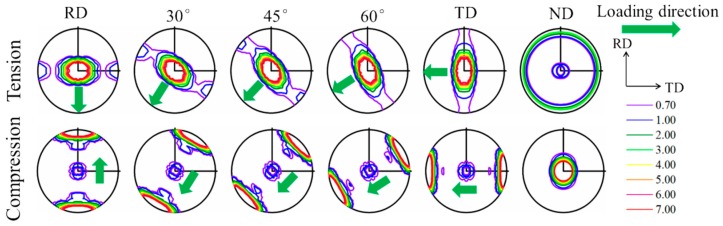
Predicted deformed texture at a strain of 15% in terms of {0002} pole figures under uniaxial tension/compression along RD, 30°, 45°, 60°, TD, and ND.

The developed textures at a strain of 15% under various loadings are shown as {0002} pole figures in Figure 7. In the case of in-plane tensions and C-ND, textures similar to the initial one develop, mainly due to deformation slips, which enhance the basal texture with c-axis perpendicular to the tensile axis or parallel to the compressive axis. Together with Figure 2, it is also indicated that the lower value of in-plane tension yield stress is associated with the larger c-axis distribution towards the loading direction. By contrast, for in-plane compression and T-ND, drastically different textures are developed owing to the extensive twinning. The peaks in the pole figures are either with c-axes reoriented parallel to the compressive loading direction or perpendicular to the tensile loading axis. The majority of the texture components are with c-axis perpendicular to the compressive loading, and the yielding stresses of in-plane compressions are almost the same.

### 4.3. R-Value Evolution

The plastic strain ratio (R-value) is an important parameter for evaluating the formation performance of metal sheets. Here, the R-value is defined as $R=\varepsilon_{22}/\varepsilon_{33}$, with axes 2 and 3 the lateral directions within the sheet plane and sheet plane normal, respectively [16]. As previously mentioned, R-values are often used to calculate material parameters involved in anisotropic yield functions. These anisotropic yield functions are such that the R-value is often assumed to be constant with strain. This assumption, although not necessary, is reasonable in the yield functions designed for face centered cubic (FCC) and body centered cubic (BCC) polycrystalline sheets because variations in R-values with applied deformation are relatively small [48].

**Figure 8:**
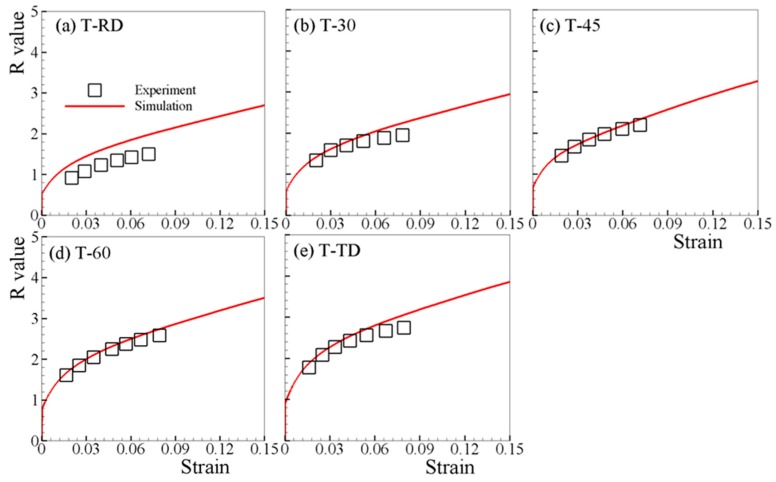
Measured and predicted R-values under (**a**) T-RD, (**b**) T-30, (**c**) T-45, (**d**) T-60, and (**e**) T-TD.

**Figure 9:**
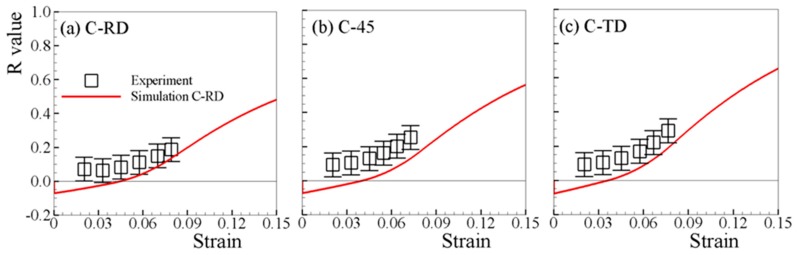
Measured and predicted R-values under (**a**) C-RD, (**b**) C-45, and (**c**) C-TD.

**Figure 10:**
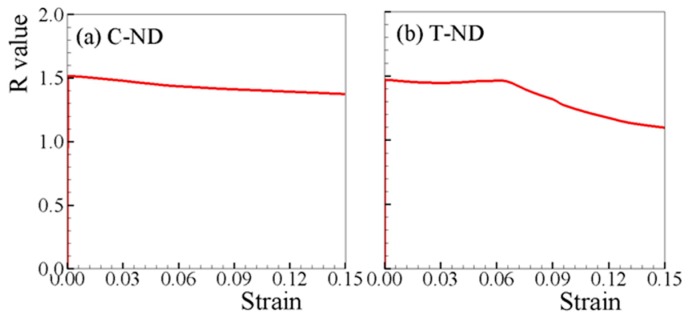
Predicted R-values under (**a**) C-ND and (**b**) T-ND.

The measured and predicted R-values under both tension and compression are respectively shown in Figure 8 and Figure 9. Since the uncertainty of the R-value measured at low strain (<2%) is too high, only the experimental results beyond strain of 2% are available [6,7]. The predicted R-values under in-plane tensions, which increase monotonically with straining, matches the experimental ones well (Figure 8). For in-plane compressions, though slightly lower than the experimental R-values, the predicted R-values are in reasonable agreement with the experimental ones (Figure 9). Figure 10a,b depict the simulated R-values associated with loading along ND, where both positive values are obtained for T-ND and C-ND. Therefore, negative R-value is not necessarily developed when twinning is active, e.g., T-ND.

**Figure 11:**
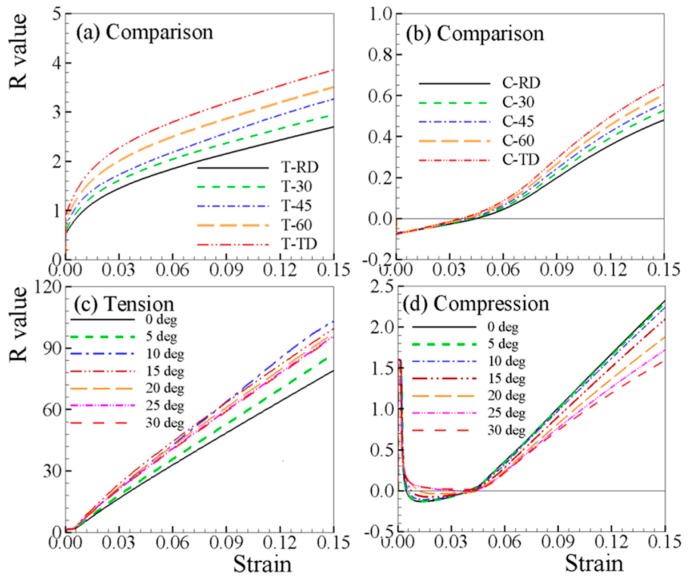
Predicted R-values under (**a**) tension and (**b**) compression of AZ31B sheet, and (**c**) tension and (**d**) compression of a single crystal.

Figure 11 compares the predicted R-values along different loading paths, where the R-value evolves significantly with straining under tension (Figure 11a). The variation in R-value with imposed tensile straining is confirmed by the experimental works on magnesium alloys at room temperature [11,49,50,51,52,53]. It is found that the predicted R-values under in-plane compressions is negative at small strain (Figure 11b). For isotropic materials and anisotropic FCC and BCC sheet metals under uniaxial compression, the lateral strains are both positive. However, in the AZ31B sheet under uniaxial compression, negative $\varepsilon_{22}$ and positive $\varepsilon_{33}$ are obtained at small strains when tensile twinning occurs. At large strains, where twinning is nearly exhausted, the predicted R-value becomes positive because the two lateral strains are both tensile. The predicted negative R-value under compression at small strains has been experimentally observed [21,53]

One may argue that strong evolution in R-value with deformation is mainly due to the strong texture evolution shown in Figure 7. It is clear that the assumption of constant in-plane R-value under tension and compression—for determining material constants in anisotropic yield functions for HCP polycrystalline sheets—is not appropriate.

In order to understand the evolution of the R-value of the AZ31B sheet, the R-value of typical single crystals that are embedded in the sheet is investigated. The Euler angle of the single crystal is prescribed to be (φ, 0°, 0°), with φ changing from 0 to 360°. Considering the crystal symmetry, the range (0–30°) of φ covers all possible orientations in the sheet with c-axis parallel to ND. As can be seen, more significant evolution of the R-values is obtained under both tension and compression (Figure 11c,d) in the single crystal compared to the AZ31B sheet. For tension of a single crystal (Figure 11c), the R-value does not necessarily increase when changing the direction from 0 to 30°, which is different from that of the AZ31B sheet (Figure 11a). The R-value reaches its highest value at 10° and lowest at 0°. For compression of a single crystal (Figure 11d), the R-value monotonically decreases with increasing angle. In some of the specially designed single crystals, the orientation is unchanged during straining. These results indicate that the HCP crystallographic structure is responsible for the significant evolution in R-value. As listed in Table 2, the hardening parameters of the four accounted deformation mechanisms are very different from each other, which leads to the different hardening behaviors of the deformation mechanisms. As a consequence, the ability of the single crystal deforms differently along the c-axis and the direction perpendicular to it. According to the rapid increase of the R-value during straining, the deformability along the c-axis becomes more difficult than those along in-plane directions. Close observation of Figure 11d reveals that negative R-values are obtained within the initial straining up to ~4% under compression lateral to the c-axis, which apparently attributed to the dominant deformation mechanism of twinning.

**Figure 12:**
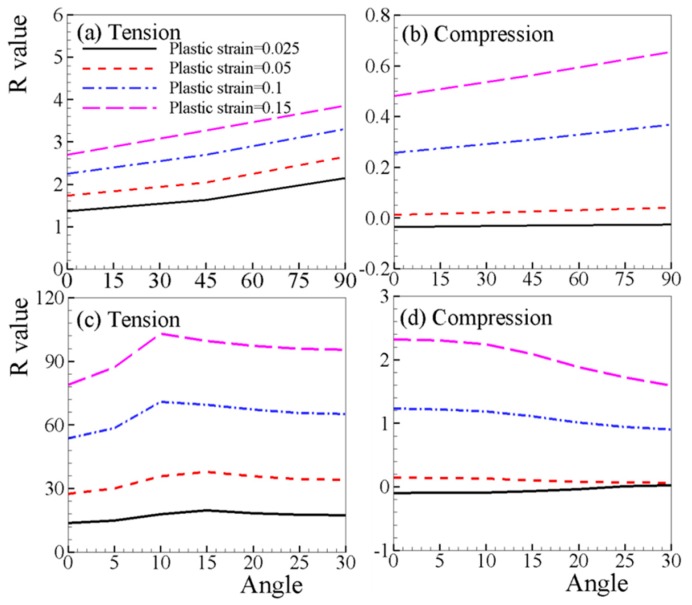
Predicted R-values of (**a**,**b**) AZ31B sheet and (**c**,**d**) single crystal with respect to loading direction.

Figure 12 shows the evolution of R-value with different loading directions at four strain levels of 0.025, 0.05, 0.1, and 0.15. When loading along RD (0°) of the sheet, the R-value is the minimum at all strain levels investigated. For the sheet, the R-value increases as the load direction changes from RD (0°) to TD (90°) under uniaxial tension. The R-value does not change significantly with respect to the loading direction when the strain level is low (ε = 0.025, 0.05), while the R-value increases at high strain levels (ε = 0.1, 0.15) (Figure 12b). Interestingly, the evolution of the R-value for single crystals is very different to that of the sheet (Figure 12c,d). The loading direction has little influence on the variation trend of R-values, and R-values only fluctuate under larger strain (ε = 0.1, 0.15). For a single crystal under compression (Figure 12d), the absolute R-value decreased as the load direction changes from the 0 (RD) to 30°, and this phenomenon is more obvious at higher strain level. The difference in the dependency of the R-value on loading direction between the sheet and the single crystal is possible since the sheet is composed of nearly 2000 single crystals whose orientations are not perfectly oriented as the single crystals studied. Therefore, in the AZ31B sheet, more deformation mechanisms may be operative than in comparison with single crystals.

## 5. Conclusions

In this paper, the anisotropy of magnesium alloy sheet has been investigated by crystal plasticity modeling. Through changing the loading directions, the stress–strain curves, relative activity of deformation mechanisms, texture development, and R-values were systematically compared. The characteristic anisotropy of Mg alloy sheets, such as tension/compression asymmetry, sinusoidal stress–strain curves when twinning is activated, negative R-value, etc., were well-captured by the model. With the aid of simulating the evolution of Mg single crystals, both the HCP crystallographic structure and the orientation distribution of the Mg sheet were responsible for the anisotropy. The polar nature of deformation twinning led to the strong tension/compression asymmetry. Moreover, twinning activity is responsible for the negative R-values under in-plane compression. These findings are in good agreement with the corresponding experimental results.

## Figures and Tables

**Table 1:** Chemical composition limits of AZ31B-0.

Al	Zn	Mn	Cu	Fe	Ni	Si	Mg
2.9	0.25	0.94	0.001	0.004	0.005	0.005	Bal

**Table 2:** List of material parameters of AZ31B sheet for viscoplastic self-consistent model and twinning and detwinning scheme (VPSC–TDT).

Mode	τ_0_ (MPa)	τ_1_ (MPa)	h_0_ (MPa)	h_1_ (MPa)	h^αβ^	A_1_	A_2_
Basal	5	7	150	25	2.0	–	–
Prismatic	95	30	350	25	1.0	–	–
Pyramidal	105	110	3000	0	1.5	–	–
Extension twin	37	0	0	0	1.0	0.65	0.55
